# Supplementary material for: Spectroscopic visualization of reversible hydrogen spillover between palladium and metal–organic frameworks toward catalytic semihydrogenation
Source: Nat Commun. 2024 Mar 22;15:2562. doi: 10.1038/s41467-024-46923-3 (PMC10959988; doi:10.1038/s41467-024-46923-3)
Supplement: Supplementary file 1 — Supplementary Information [file 41467_2024_46923_MOESM1_ESM.pdf]

Supplementary Information for

Spectroscopic visualization of reversible hydrogen spillover between palladium and metal–organic frameworks toward catalytic semihydrogenation

Qiaoxi Liu[1,2], Wenjie Xu[1], Hao Huang[1], Hongwei Shou[1], Jingxiang Low[1], Yitao Dai[1], Wanbing Gong[1], Youyou Li[1], Delong Duan[1], Wenqing Zhang[1], Yawen Jiang[1], Guikai Zhang[3], Dengfeng Cao[1], Kecheng Wei[1], Ran Long[1]*, Shuangming Chen[1], Li Song[1] and Yujie Xiong[1,2,4]*

[1]Hefei National Research Center for Physical Sciences at the Microscale, Key Laboratory of Precision and Intelligent Chemistry, School of Chemistry and Materials Science, School of Nuclear Science and Technology, National Synchrotron Radiation Laboratory, University of Science and Technology of China, Hefei, Anhui 230026, China.

[2]Suzhou Institute for Advanced Research, University of Science and Technology of China, Suzhou, Jiangsu 215123, China.

[3]Beijing Synchrotron Radiation Facility, Institute of High Energy Physics, Chinese Academy of Sciences, Beijing 100049, China.

[4]Key Laboratory of Functional Molecular Solids, Ministry of Education, Anhui Engineering Research Center of Carbon Neutrality, College of Chemistry and Materials Science, Anhui Normal University, Wuhu, Anhui 241000, China.

**e-mail: yjxiong@ustc.edu.cn; longran@ustc.edu.cn*

Contents

1. **Supplementary Methods**
2. **Supplementary Figures 1–30**
3. **Supplementary Table 1–2**
4. **Supplementary References**

1. Supplementary Methods

Chemicals. Hydrochloric acid (36-38%, AR), acetonitrile (CH_3CN, AR), chloroauric acid hydrated ($HAuCl_4·4H_2O$, AR), cetyltrimethylammonium bromide (CTAB, 99.0%), ascorbic acid (AA, 99.7%), zinc nitrate hexahydrate ($Zn(NO_3)_2·6H_2O$, 99.0%), methylbenzene (AR) and N,N-dimethylformamide (DMF, AR) were purchased from Sinopharm Chemical Reagent Co., Ltd. 2-Methylimidazole (98.0%) was purchased from Energy Chemical. 4-nitrothiophenol (pNTP, >95%) was purchased from Tokyo Chemical Industry. Potassium palladium (II) chloride (K_2PdCl_4), poly(vinyl pyrrolidone) (PVP) and sodium borohydride ($NaBH_4$) were purchased from Aladdin Industrial Inc. Silver nitrate ($AgNO_3$, 99.0%) was purchased from Sigma-Aldrich. Sodium citrate ($C_6H_5Na_3O_7$, 99.0%) was purchased from J&K Scientific. The water used in all experiments was deionized (DI). All chemicals were used as received without further purification.

Synthesis of Pd nanocubes (Pd NCs). The Pd NCs were synthesized by following a reported method. 105 mg PVP, 60 mg L-ascorbic acid, and 600 mg KBr were dissolved in 8 mL DI water. The solution was heated at 80 °C for 15 min under magnetic stirring before 3 mL aqueous solution of K_2PdCl_4 (57 mg) was added. The solution was maintained at 80 °C for 3 h. The Pd NCs were collected by centrifugation for three times using water, and the precipitation was redispersed in DI water.

Synthesis of Pd NCs/ZIF-8 (Pd/ZIF-8 for NMR) composite structures. Typically, a 1-mL aqueous suspension of Pd NCs (1 mg/mL) was mixed with a 50-mL methanol solution of 2-methylimidazole (102.5 mg) and a 50-mL methanol solution of $Zn(NO_3)_2·6H_2O$ (372 mg), which was then kept undisturbed at 0 °C for 10 min. The products were collected by centrifugation and washed twice with methanol. The precipitation was further dried overnight at 60 °C under vacuum condition. The resulted Pd/ZIF-8 sample is an ideal system for NMR investigation whose surface of Pd is enclosed by Pd(100) facets and has abundant Pd–ZIF-8 interface, which is beneficial for examining hydrogen spillover of Pd-ZIF-8 interface by solid H^2 NMR.

Synthesis of Au nanorods. The Au nanorods were synthesized by following a reported seed-mediated growth method[2]. The suspension of the Au nanorods was centrifuged three times at 8,000 rpm for 10 min. The precipitation was redispersed in DI water, and the concentration of the Au nanorods was 1 mg mL^{-1} which was measured by inductively coupled plasma–mass spectrometry (ICP–MS).

Synthesis of Pd clusters/ZIF-8 (Pd/ZIF-8 for XRD) composite structures. To synthesize the Pd clusters/ZIF-8 composite structures, the ZIF-8 matrix was prepared firstly. 1.467 g $Zn(NO_3)_2·6H_2O$ and 3.245 g 2-methylimidazole were dissolved in 100 mL methanol separately. These two solutions were then mixed, and kept in an oil bath at 50 °C and stirred at 300 rpm for 12 h. The products were washed by methanol for 3 times and centrifuged at 8,000 rpm for 10 min. The ZIF-8 white powder was obtained

under heat treatment in vacuum for 12 h. 1 g ZIF-8 was dispersed in water under 300 rpm. 0.5 mL K_2PdCl_4 solution (0.1 M) was added into the solution and stirred for 12 h for well blend. 0.2 g $NaBH_4$ was added into the solution slowly. The products were washed with water for 3 times and centrifuged at 8,000 rpm for 10 min. The brown powder was obtained under heat treatment in vacuum for 12 h. The sample of Pd/ZIF-8 for XRD was used for synchrotron radiation-based XRD because the diffraction of Pd lattice is weak so as to have little effect on the diffraction of ZIF-8.

Sample characterizations. Prior to electron microscopy characterizations, a drop of the aqueous suspension of particles was placed on a piece of carbon-coated copper grid or silicon wafer and dried under ambient conditions. Transmission electron microscopy (TEM), high-resolution TEM (HRTEM), scanning TEM (STEM) images and energy-dispersive X-ray spectroscopy (EDS) analyses were taken on a JEOL JEM-2100F field-emission high-resolution transmission electron microscope operated at 200 kV. Scanning electron microscopy (SEM) images were taken on a FEI Sirion-200 field emission scanning electron microscope operated at 5 kV. Powder X-ray diffraction (XRD) patterns were recorded by using a Philips X'Pert Pro Super X-ray diffractometer with Cu-Kα radiation (λ = 1.5418 Å). The XRD patterns of samples under hydrogen condition were recorded by placing samples in quartz tube with 100% H_2 at 101 kPa at 293 K. High-resolution XRD patterns for thin films were recorded by using a X `Pert3 MRD with Cu-Kα radiation (λ = 1.5418 Å). UV−vis extinction spectra were recorded on an Agilent Technologies Cary 60 spectrometer. The concentrations of elements were measured with a Thermo Scientific Plasma Quad 3 ICP−MS after dissolving them with a mixture of HCl and HNO_3 (3:1, volume ratio). Focused ion beam (FIB) was performed on a FEI, Helios NanoLab650. The potential presence of mesopores was recorded by using Micromeritics ASAP 2460.

Synchrotron radiation-based XRD. XRD patterns of Pd clusters/ZIF-8 were recorded using six-circle diffractometer (Huber 5021) in the air and H_2 atmosphere at beamline BL14B of SSRF with a step scan of 0.02°. XRD patterns of Au/Pd/ZIF-8 were recorded using six-circle diffractometer (Huber 5020) in the air and H_2 atmosphere at beamline 4B9A at BSRF with a step scan of 0.02°.

Solid-state ^{2}H NMR measurements. Solid-state ^{2}H nuclear magnetic resonance (NMR) spectra were measured at 293 K with a frequency of 15 MHz using a Bruker AVANCE AV400 NMR spectrometer. Samples were evacuated in vacuum at 333 K for 12 h. After evacuation, the samples were sealed in quartz tube with 100% D_2 gas at 101 kPa at 293 K.

Determination of turnover frequency (TOF) of pNTP hydrogenation.

According to Van Hardeveld's study, cuboctahedron is approximately equivalent to the sphere as for nanoparticle[3]. Thus, the total number of atoms (N_T) was defined as follows:

$$N_T = 16m^3 - 33m^2 + 24m - 6$$

The number of surface atoms (N_S):

$$N_S = 30m^2 - 60m + 32$$

where m represents the number of atoms lying on an equivalent edge (corner atoms included). As such, the surface ratio of Au atoms:

$$\text{ratio}_S = \frac{N_S}{N_T}$$

On the basis of geometric principle,

$$m = \frac{d_{Au\ particle}}{\sqrt{10} \times 2r_{Au\ atom}}$$

where $d_{Au\ particle}$ represents the diameter of Au particle and $r_{Au\ atom}$ represents the section radius of Au atom. $d_{Au\ particle}$ is *ca.* 45.0 nm, and $r_{Au\ atom}$ is *ca.* 0.144 nm. In this case, the ratio_S can be calculated to be about 0.0380. The TOF was calculated by the following equation:

$$\text{TOF} = \frac{mol_{product}}{\text{ratio}_S \times mol_{total\ Au} \times time}$$

where $mol_{product}$ is the number of pATP. TOF values were calculated for cases with less than 20% conversion rate, where the substrate was in excess of the balanced endpoint. The results of TOF for different samples can be found in Supplementary Table 2.

Theoretical calculation. The theoretical calculations of hydrogen spillover were carried out using the Vienna ab initio simulation package (VASP)[4] within the projector augmented wave (PAW)[5]. The Perdew-Burke-Ernzerh (PBE) functional was applied within the Generalized Gradient Approximation (GGA)[6]. Only the gamma point of the Brillouin zone was used in all simulations. The structure optimization force and energy were minimized below 0.05 eV·Å and 10^{-6} eV, respectively. The cut-off energy for optimization was 450 eV. Pd (100) was used as the sites where H_2 was dissociated to hydrogen atoms and H_{ad} atoms spilled over to ZIF-8. ZIF-8 was captured by Zn atoms combined with 5 × 5 Pd (100) lattice. To shield mirror interactions, a vacuum along c direction with a thickness of 13 Å was performed. The computational formula of H_{ad} adsorption energy is $E_{ad} = (E_{tot} - E_{slab} - \frac{1}{2}nE_{H_2})/n$, where E_{tot} and E_{slab} represent the energy of Pd or Pd/MOF with or without H atom, respectively, and n represents the number of hydrogen atoms. The constrained ab initio molecular dynamic (cAIMD) within a slow-growth[7] was utilized to reveal the potential barriers and sample the free energy profile. This method allowed us to change the reaction coordinate along the reaction path for the rare reaction event by garnering a holonomic constraint. The free energy ΔA(ξ) can be integrated according to equation:

$$\Delta A(\xi) = -\int_{\xi 0}^{\xi} d\xi' \langle\, f(\xi')\,\rangle_{\xi'}$$

where ξ' is the specified reaction coordinate, and $\langle fs(\xi')\rangle\xi'$ represents the average force of constraint. The simulations were performed under 300 K with a Nose–Hoover thermostat. Two atomic mass units were adopted for all H atoms with a time step of 1.0 fs[8,9]. For the free energy profile of hydrogen spillover from Pd to ZIF-8, the collective variable (CV) (C-H distance) increment is set to 0.0004 Å and the simulation time is set to 6.5 ps. For the free energy profile of hydrogen spillover from ZIF-8 back to Pd, CV is set to 0.00003 and simulation time is set to 5 ps. The detailed setting for hydrogen

spillover is shown in Supplementary Fig. 27.

Determination of TOF of alkynes hydrogenation. The volume of Pd shell:

$$V_{shell} = \pi r^2{}_{Au/Pd} h_{Au/Pd} - \pi r^2{}_{Au\ NRs} h_{Au\ NRs}$$

where r and h represent the section radius and length of nanorods, respectively.
The surface area of Pd shell:

$$S_{shell} = 2\pi r^2{}_{Au/Pd} + 2\pi r_{Au/Pd} h_{Au/Pd}$$

According to HRTEM images of Au/Pd, the Pd shell is dominated by (100) lattice. As such, the surface ratio of Pd atoms:

$$\text{ratio}_{shell} = \frac{2S_{shell}}{a^2{}_{Pd}} / \frac{4V_{shell}}{a^3{}_{Pd}}$$

The unit cell parameter a_{Pd} = ca. 0.194 nm. In this case, the ratio_{shell} is 0.0395.
The TOF was calculated by the following equation:

$$\text{TOF} = \frac{mol_{product}}{\text{ratio}_{shell} \times mol_{total\ Pd} \times time}$$

where $mol_{product}$ is the number of alkyne hydrogenation products—alkene and alkane.

We calculated the TOF in the case of less than 50% conversion rate where substrate was much more than the balanced endpoint.

2. Supplementary Figures

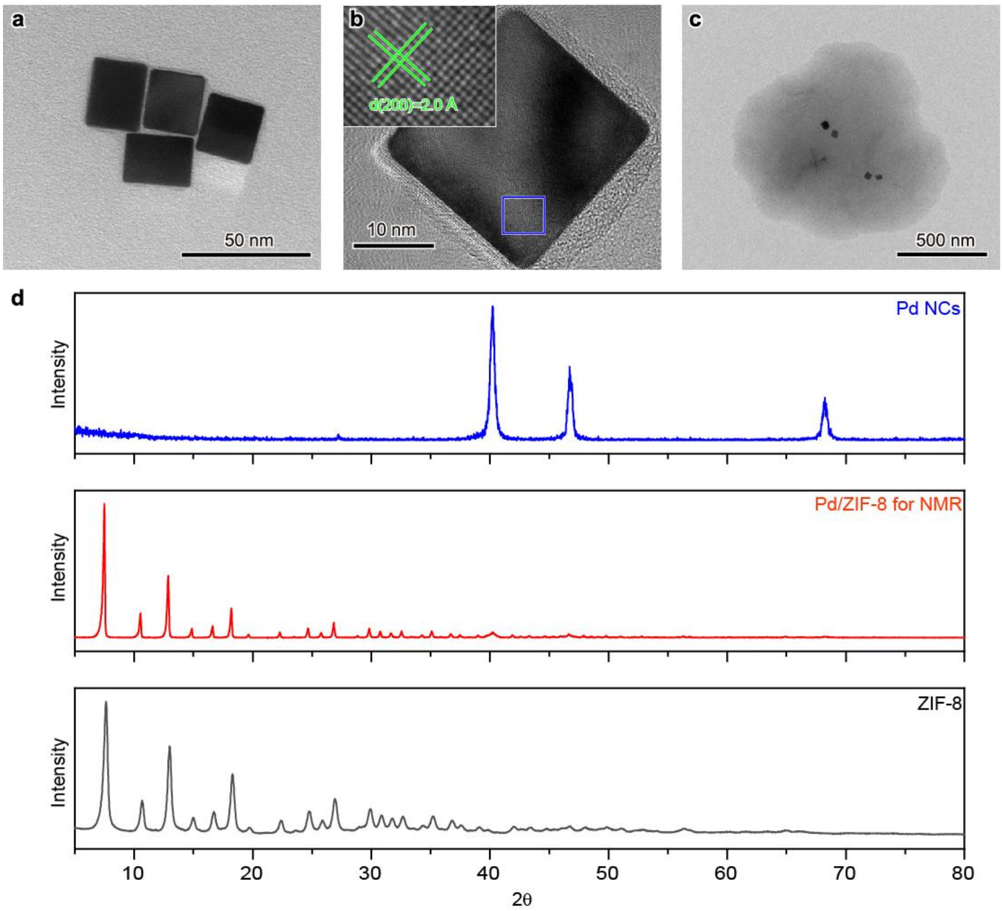

Supplementary Fig. 1 | Characterization of Pd NCs and Pd/ZIF-8 for NMR. (**a**) TEM image of Pd NCs. (**b**) HRTEM images of Pd NCs. (**c**) TEM image of Pd/ZIF-8 for NMR. (**d**) XRD patterns of Pd NCs, Pd/ZIF-8 for NMR and ZIF-8.

According to HRTEM images (Supplementary Fig. 1b), the surface of Pd NCs is dominated by Pd(100) facets.

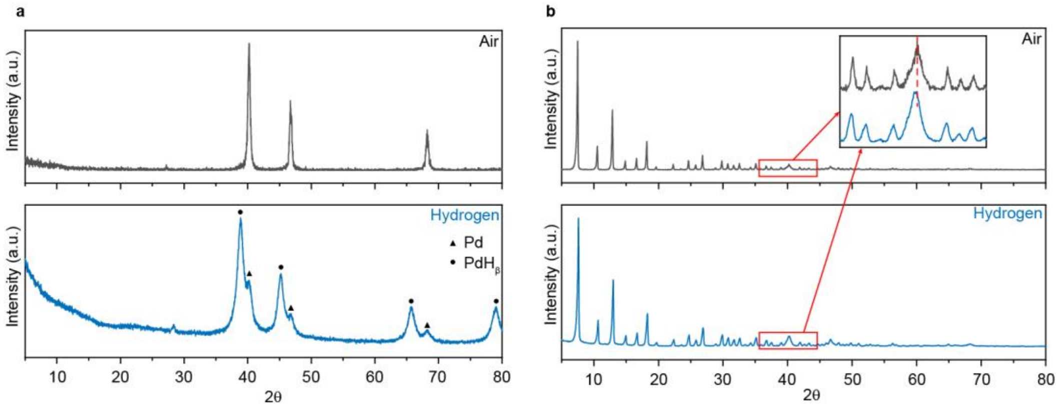

Supplementary Fig. 2 | XRD characterization. XRD patterns of (a) Pd NCs and (b) Pd/ZIF-8 for XRD under air and H_2 conditions.

As illustrated by the XRD patterns of Pd NCs and Pd/ZIF-8 under air and H_2 conditions, the diffraction peak of PdH_β appears at 38.9° for both Pd NCs and Pd/ZIF-8.

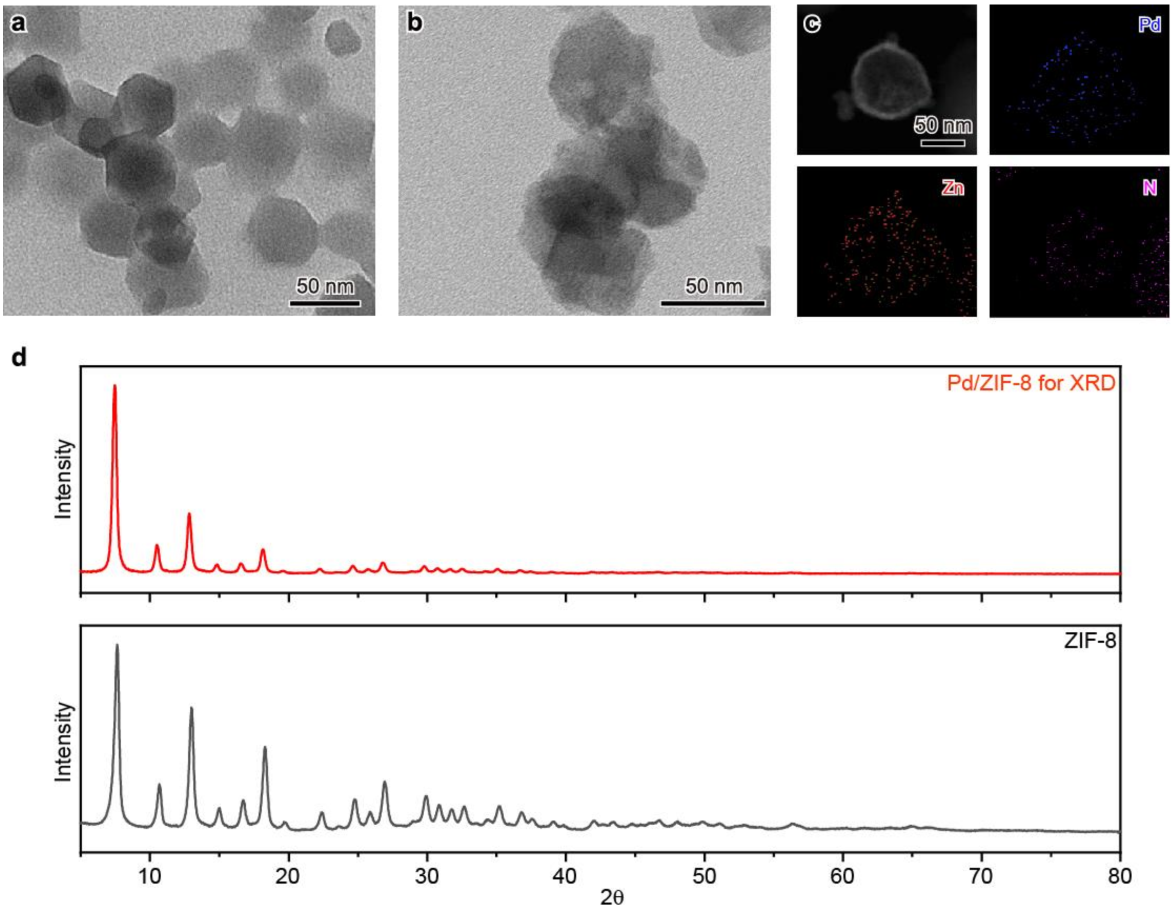

Supplementary Fig. 3 | Characterization of ZIF-8 and Pd/ZIF-8 for XRD. (**a**) TEM image of ZIF-8. (**b**) TEM image of Pd/ZIF-8 for XRD. (**c**) EDS mapping of Pd/ZIF-8 for XRD. (**d**) XRD patterns of Pd/ZIF-8 for XRD and ZIF-8.

According to EDS mapping (Supplementary Fig. 3c) and XRD patterns (Supplementary Fig. 3d), Pd in the sample of Pd/ZIF-8 for XRD exists in the form of clusters.

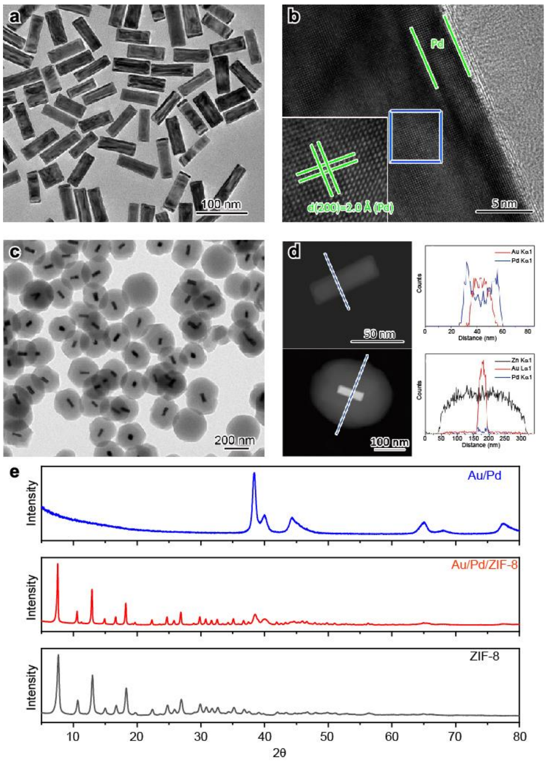

Supplementary Fig. 4 | Characterization of Au/Pd/ZIF-8. (**a**) TEM and (**b**) HRTEM images of Au/Pd core–shell structure recorded along [001] orientation. (**c**) TEM image of Au/Pd/ZIF-8. (**d**) STEM image and EDS line scan profiles of Au/Pd and Au/Pd/ZIF-8. (**e**) XRD patterns of ZIF-8 and Au/Pd/ZIF-8.

As indicated by HRTEM image (Supplementary Fig. 4b), the resultant nanostructures are enclosed by (100) facets. The lattice fringes with a period of 2.0 Å can be well assigned to the (200) spacing of face-centered cubic (*fcc*) Pd.

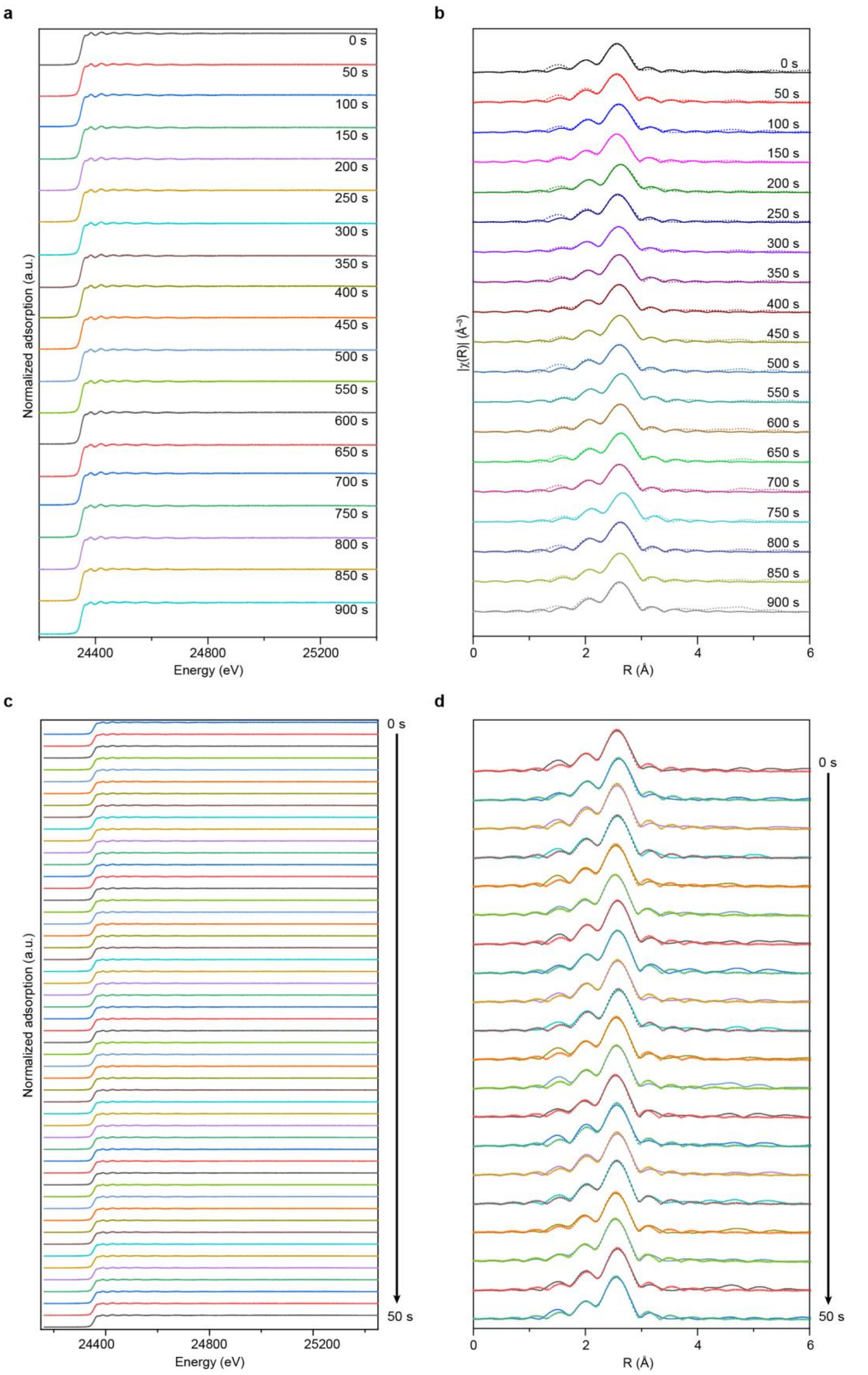

Supplementary Fig. 5 | EXAFS results of Au/Pd/ZIF-8-4% H_2. (**a**) Series of time-resolved Pd K-edge EXAFS spectra (50 s time resolution). (**b**) Series of time-resolved Pd K-edge k^2-weighted EXAFS Fourier transforms at $k = 3.0{\sim}11.1$ Å^{-1} (50 s time resolution) (solid lines) and fitted Fourier-

filtered EXAFS spectra in R-space (50 s time resolution) (dashed lines). (**c**) Series of time-resolved Pd K-edge EXAFS spectra (1 s time resolution). (**d**) Series of time-resolved Pd K-edge k^2-weighted EXAFS Fourier transforms at $k = 3.0{\sim}11.1$ Å^{-1} (2.5 s time resolution) (solid lines) and fitted Fourier-filtered EXAFS spectra in R-space (2.5 s time resolution) (dashed lines). Spectra are shifted vertically for clarity.

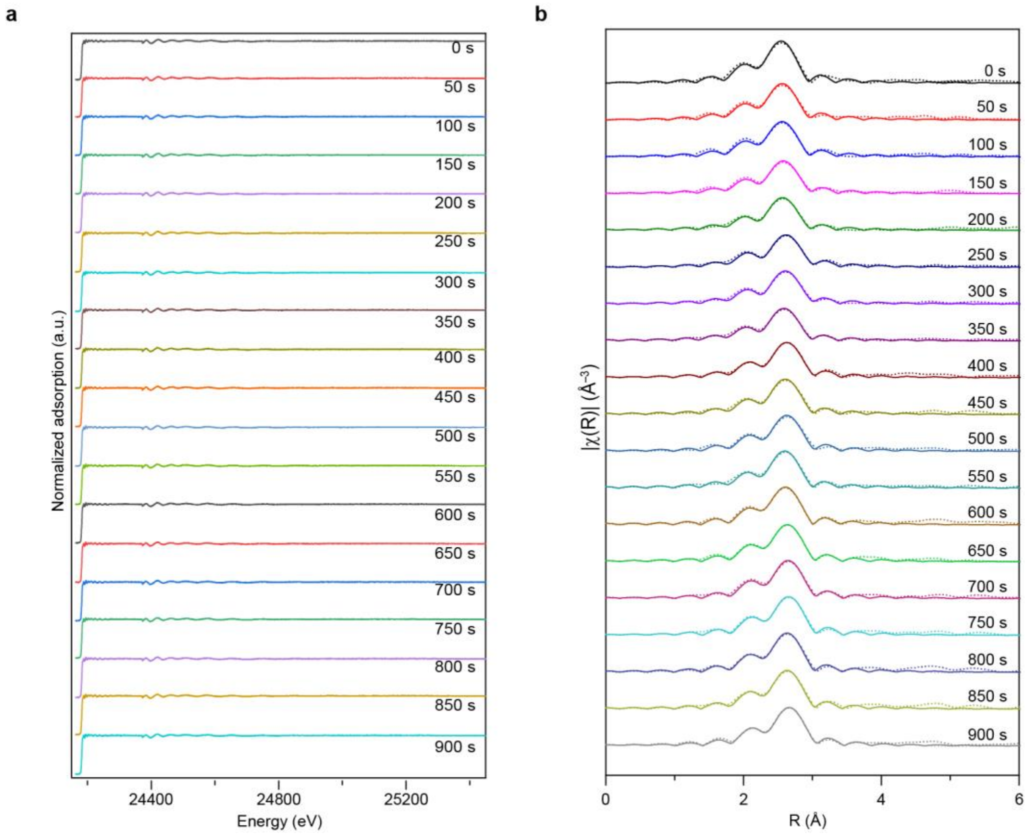

Supplementary Fig. 6 | EXAFS results of Au/Pd/ZIF-8-100% H_2. (**a**) Series of time-resolved Pd K-edge EXAFS spectra (50 s time resolution). (**b**) Series of time-resolved Pd K-edge k^2-weighted EXAFS Fourier transforms at $k = 3.0$~11.1 $Å^{-1}$ (50 s time resolution) (solid lines) and fitted Fourier-filtered EXAFS spectra in R-space (50 s time resolution) (dashed lines). Spectra are shifted vertically for clarity.

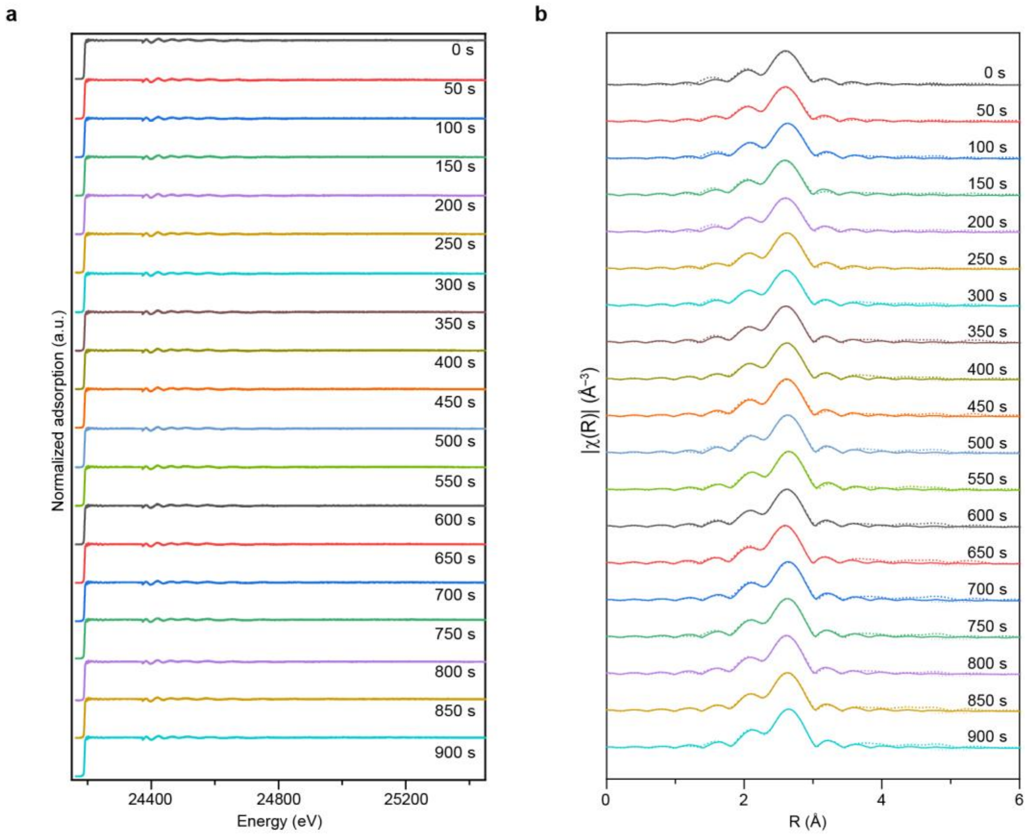

Supplementary Fig. 7 | EXAFS results of Au/Pd-4% H_2. (**a**) Series of time-resolved Pd K-edge EXAFS spectra (50 s time resolution). (**b**) Series of time-resolved Pd K-edge k^2-weighted EXAFS Fourier transforms at $k = 3.0 \sim 11.1$ Å^{-1} (50 s time resolution) (solid lines) and fitted Fourier-filtered EXAFS spectra in R-space (50 s time resolution) (dashed lines). Spectra are shifted vertically for clarity.

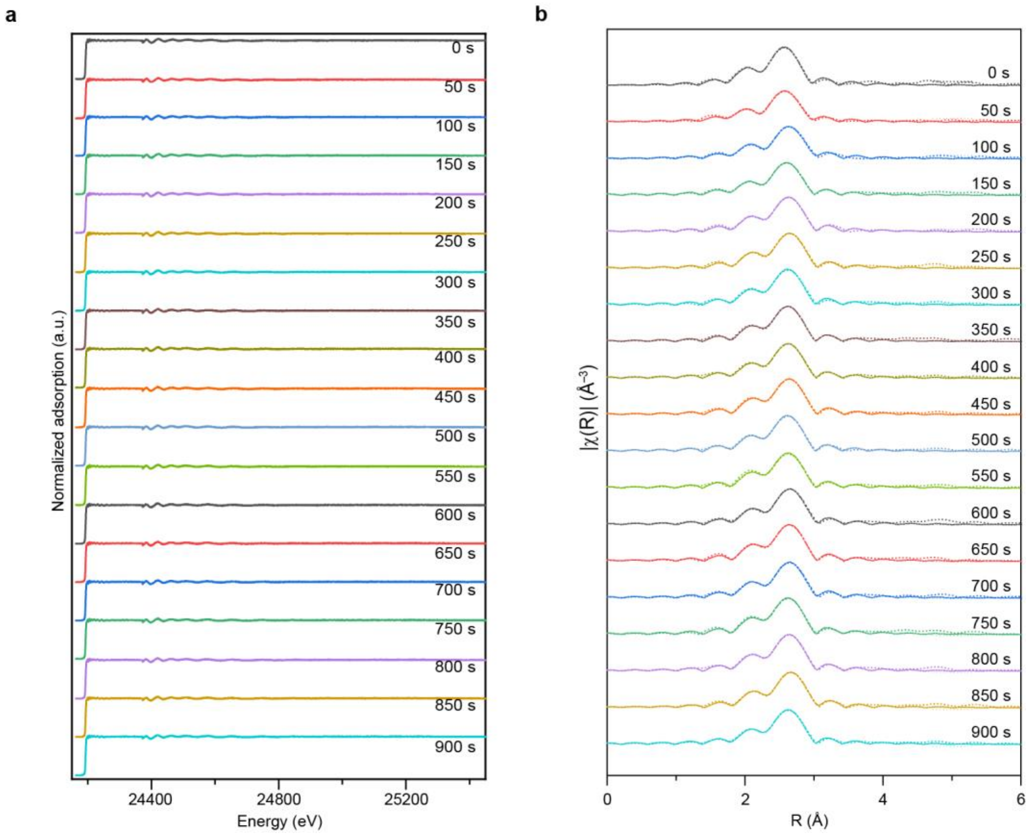

Supplementary Fig. 8 | EXAFS results of Au/Pd-100% H_2. (**a**) Series of time-resolved Pd K-edge EXAFS spectra (50 s time resolution). (**b**) Series of time-resolved Pd K-edge k^2-weighted EXAFS Fourier transforms at $k = 3.0$~11.1 Å^{-1} (50 s time resolution) (solid lines) and fitted Fourier-filtered EXAFS spectra in R-space (50 s time resolution) (dashed lines). Spectra are shifted vertically for clarity.

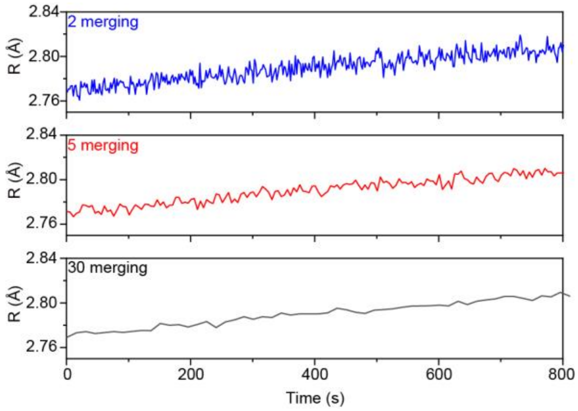

Supplementary Fig. 9 | EXAFS results of Au/Pd/ZIF-8-4% H_2 by different merging.

As demonstrated by Supplementary Fig. 9, the 5-merging spectrum shows clear details and has a good ratio of signal to noise (S/N). This indicates that merging 5 spectra is a desirable method for processing the data.

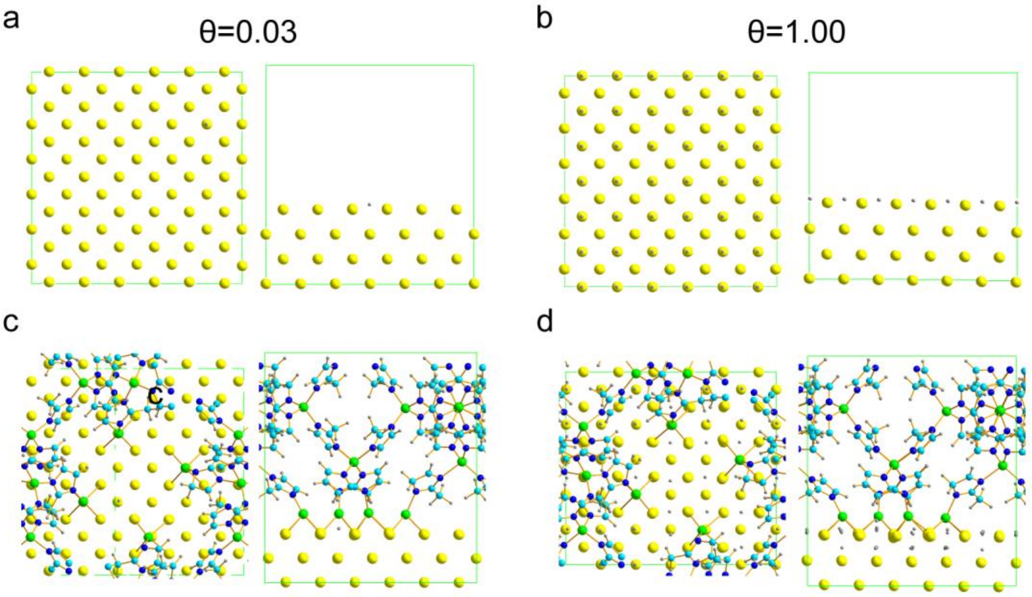

Supplementary Fig. 10 | Structures of PdH and PdH-ZIF-8 from top view (left) and side view (right) with H_{ad} coverage (θ) of 0.03 and 1.00. (**a**) Structure of $PdH_{\theta=0.03}$. (**b**) Structure of $PdH_{\theta=1.00}$. (**c**) Structure of $PdH_{\theta=0.03}$-ZIF-8. (**d**) Structure of $PdH_{\theta=1.00}$-ZIF-8. Pd: golden; H: grey; Zn: green; C: light blue; N: navy blue.

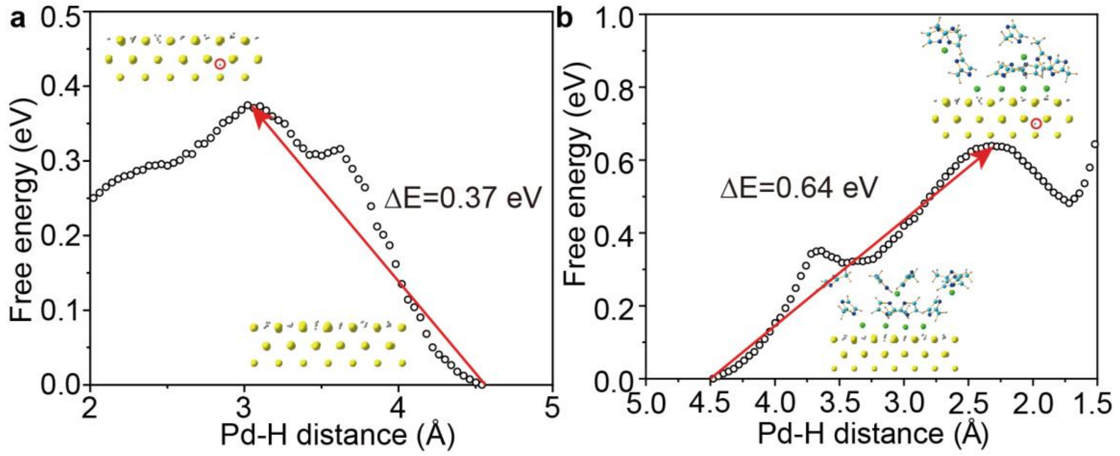

Supplementary Fig. 11 | Free energy profiles of hydrogen atoms migrating from Pd surface to octahedral sites in Pd or Pd/ZIF-8. (**a**) Free energy profiles of hydrogen atoms migrating from Pd surface to octahedral sites in Pd. (**b**) Free energy profiles of hydrogen atoms migrating from Pd surface to octahedral sites in Pd/ZIF-8.

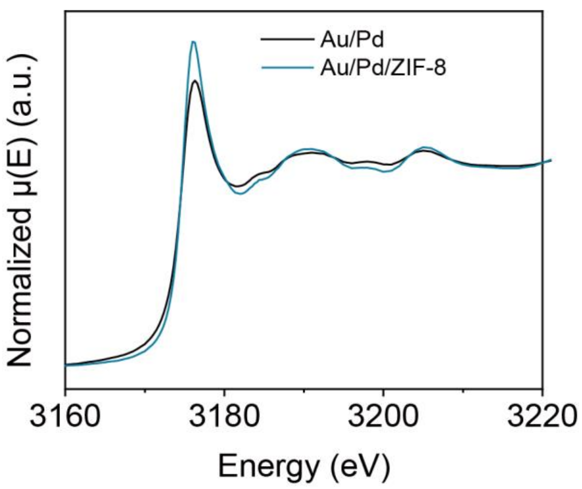

Supplementary Fig. 12 | Pd L_3-edge X-ray absorption spectroscopy (XAS) of Au/Pd and Au/Pd/ZIF-8.

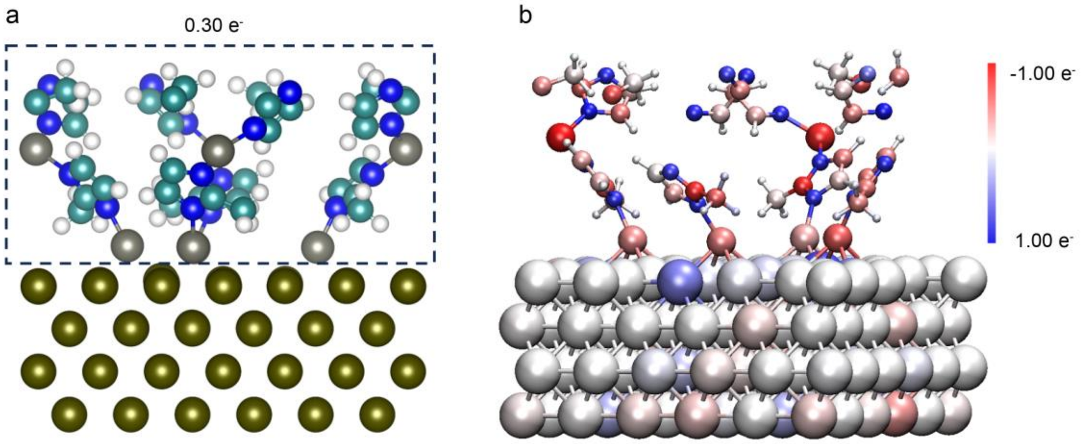

Supplementary Fig. 13 | The charge distribution of Pd–ZIF-8. (a) Bader analysis of Pd–ZIF-8. (b) Atomic coloring diagram of Pd–ZIF-8. Blue color corresponds to an electron gain, while red color indicates an electron loss.

Upon conducting Bader charge analysis, it is revealed that ZIF-8 receives 0.3 e^-, indicative of a slight transfer of 4d-electrons from Pd to ZIF-8. Insights drawn from the atomic color map suggest that the transfer of electrons occurs predominantly from Pd to N within ZIF-8.

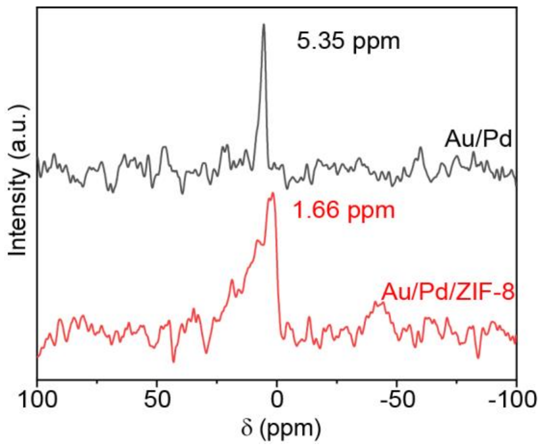

Supplementary Fig. 14 | Solid-state ^{2}H NMR spectra for Au/Pd/ZIF-8 exposed to D_2, in reference to Au/Pd.

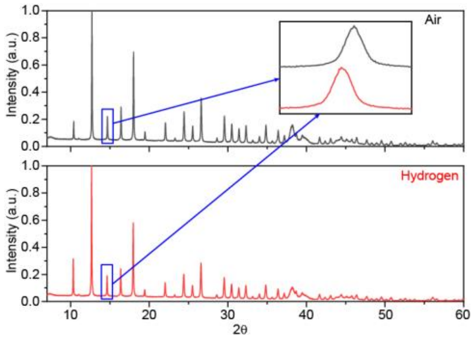

Supplementary Fig. 15 | Synchrotron radiation-based XRD patterns of Au/Pd/ZIF-8 before and after exposed to H_2.

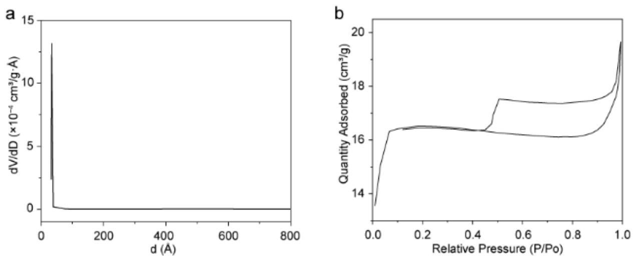

Supplementary Fig. 16 | The channel structure of Pd/ZIF-8 composite structure. (**a**) Pore size distribution and (**b**) N_2 adsorption-desorption isotherms of ZIF-8.

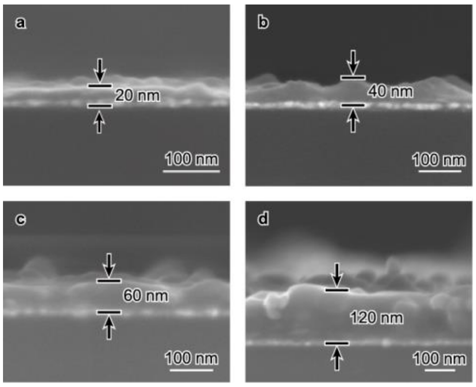

Supplementary Fig. 17 | Cross-sectional SEM characterization of Pd/ZIF-8 composite structure. Cross-sectional SEM images of (**a**) Pd/ZIF-8-20 nm, (**b**) Pd/ZIF-8-40 nm, (**c**) Pd/ZIF-8-60 nm, and (**d**) Pd/ZIF-8-120 nm.

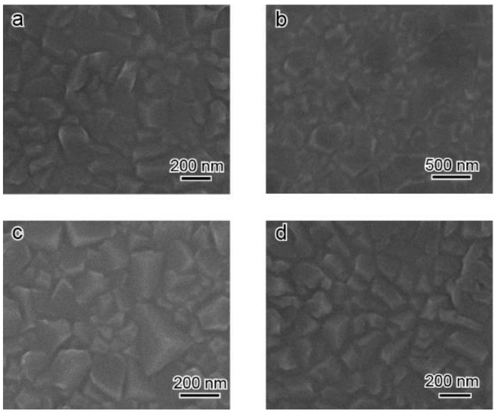

Supplementary Fig. 18 | SEM characterization of Pd/ZIF-8 composite structure. SEM images of (**a**) Pd/ZIF-8-20 nm, (**b**) Pd/ZIF-8-40 nm, (**c**) Pd/ZIF-8-60 nm, and (**d**) Pd/ZIF-8-120 nm.

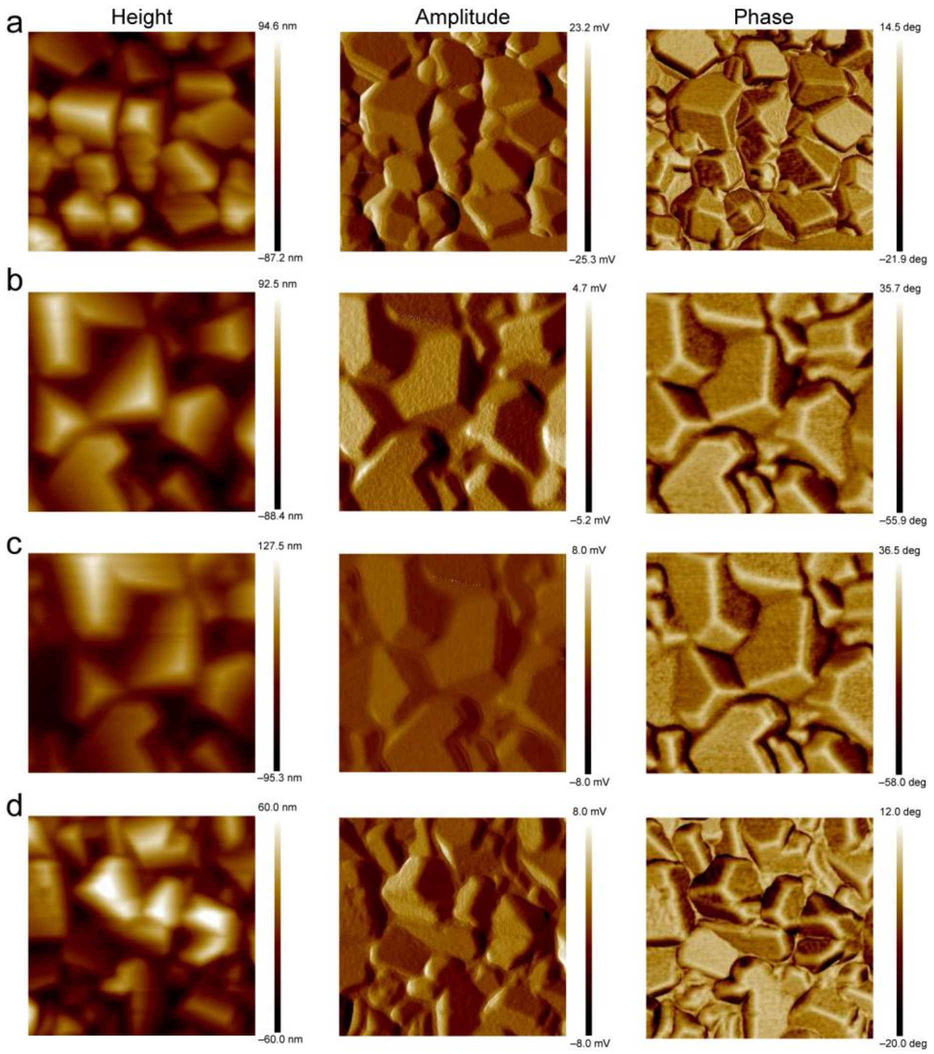

Supplementary Fig. 19 | AFM characterization of Pd/ZIF-8 composite structure. AFM analysis (1×1 μm) of ZIF-8 films with different thicknesses: (**a**) 20 nm, (**b**) 40 nm, (**c**) 60 nm, and (**d**) 120 nm.

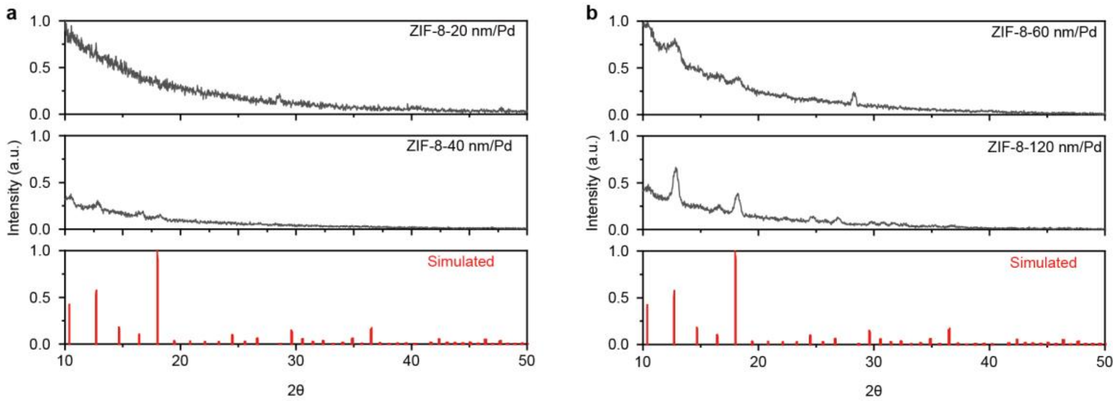

Supplementary Fig. 20 | XRD characterization of Pd/ZIF-8 composite structure. XRD patterns of (**a**) Pd/ZIF-8-20 nm and Pd/ZIF-8-40 nm, (**b**) Pd/ZIF-8-60 nm, and Pd/ZIF-8-120 nm.

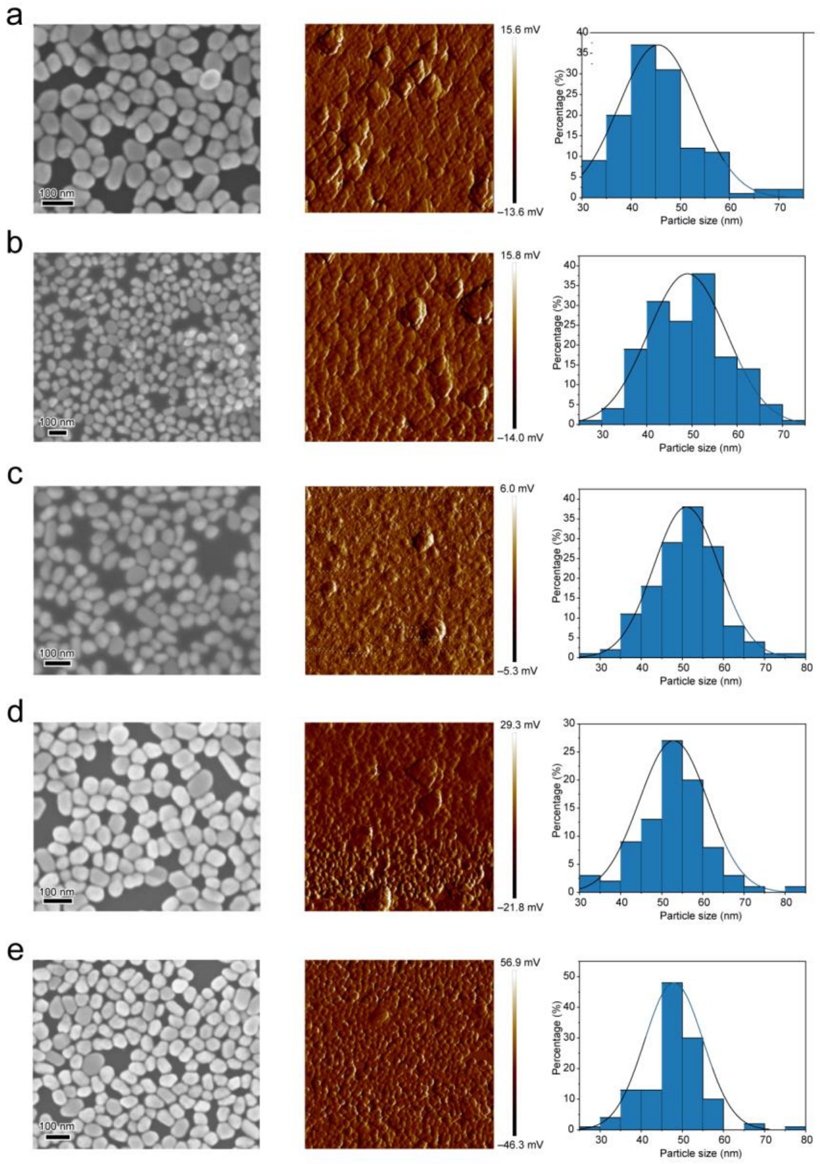

Supplementary Fig. 21 | SEM images, AFM analysis (2×2 μm) and particle size distribution of Au particles. (**a**) Pd/Au, (**b**) Pd/ZIF-8-20 nm/Au, (**c**) Pd/ZIF-8-40 nm/Au, (**d**) Pd/ZIF-8-60 nm/Au, and (**e**) Pd/ZIF-8-120 nm/Au.

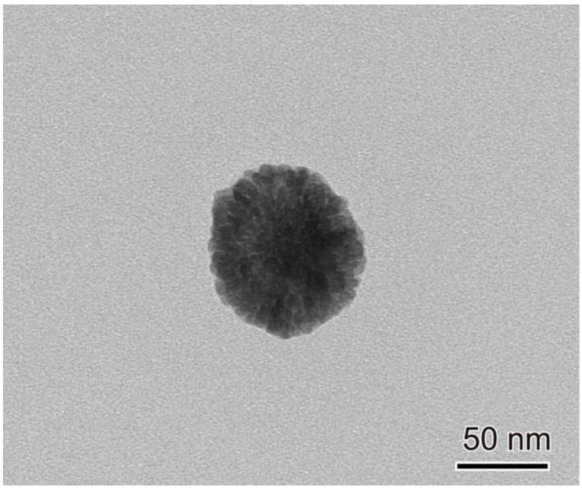

Supplementary Fig. 22 | TEM image of Au nanoparticles.

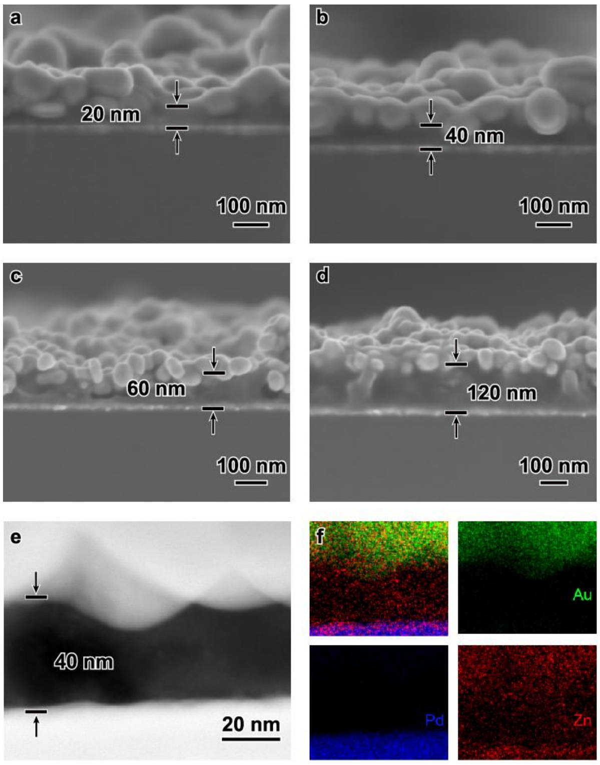

Supplementary Fig. 23 | SEM images and element analysis of Pd/ZIF-8/Au. Cross-sectional SEM images of (**a**) Pd/ZIF-8-20 nm/Au, (**b**) Pd/ZIF-8-40 nm/Au, (**c**) Pd/ZIF-8-60 nm/Au, and (**d**) Pd/ZIF-8-120 nm/Au. (**e**) Cross-sectional TEM image of Pd/ZIF-8-40 nm/Au. (**f**) EDS elemental mapping analysis of Pd/ZIF-8-40 nm/Au.

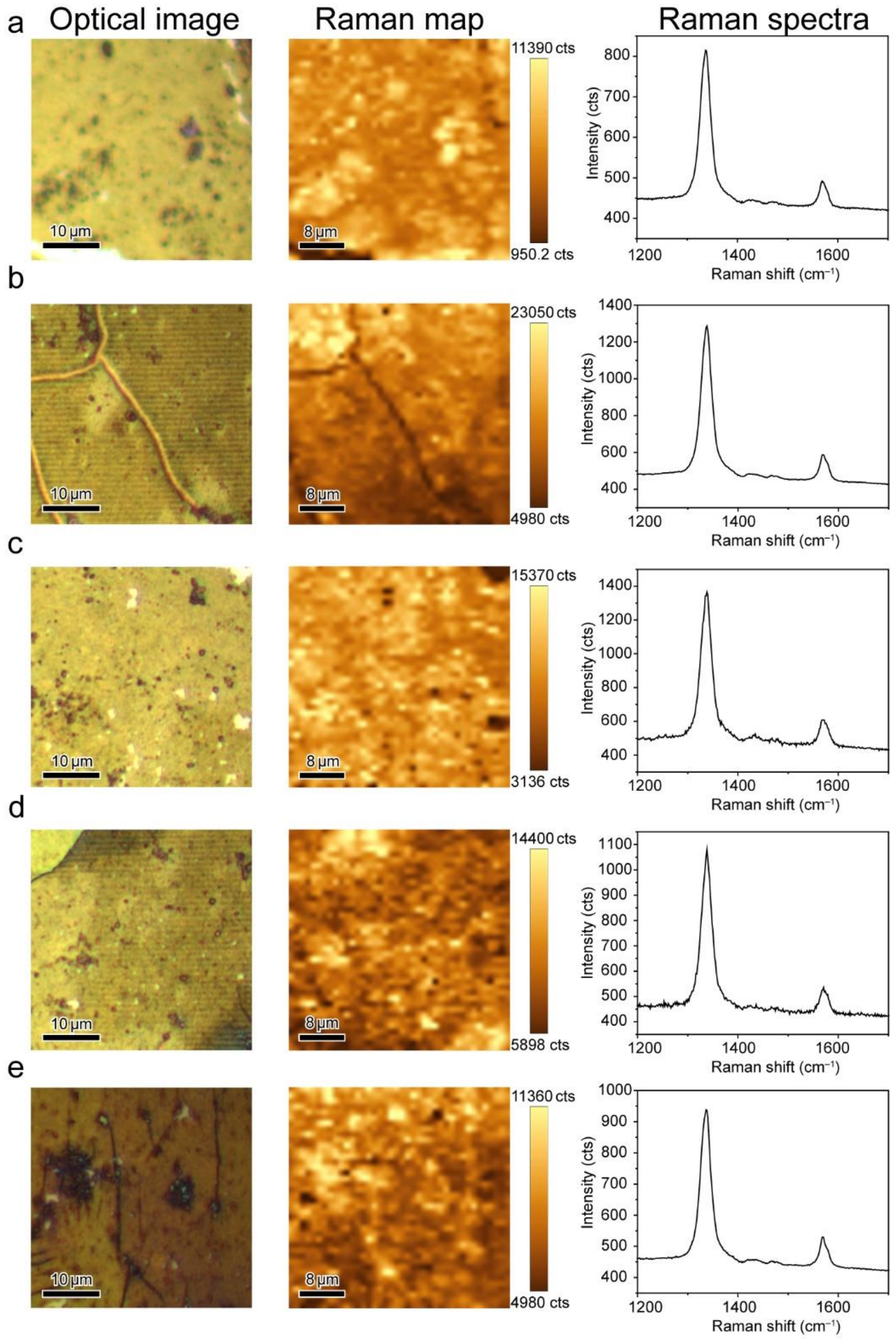

Supplementary Fig. 24 | Raman imaging of Pd/ZIF-8/Au. Raman imaging of Au particles of (**a**) Pd/Au, (**b**) Pd/ZIF-8-20 nm/Au, (**c**) Pd/ZIF-8-40 nm/Au, (**d**) Pd/ZIF-8-60 nm/Au, and (**e**) Pd/ZIF-8-120 nm/Au. The Raman map uses the peak area from 1300 to 1370 cm^{-1} for image formation.

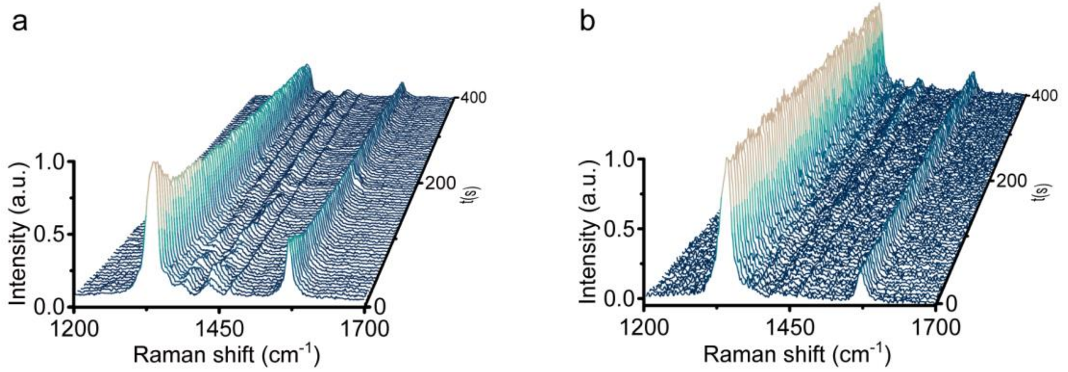

Supplementary Fig. 25 | *In situ* SERS spectra of pNTP hydrogenation. *In situ* SERS spectra of pNTP hydrogenation on (**a**) Pd/Au and (**b**) Pd/Au@SiO_2.

In situ SERS spectra of pNTP hydrogenation on Pd/Au structures illustrate that pNTP can be transformed to pATP with a higher rate compared to Pd/ZIF-8-x nm/Au. Au@SiO_2 is synthesized by Liz-Marzán's method which has porous silica shells[10]. Supplementary Figure 25b demonstrates that pNTP cannot be hydrogenated on the Pd/Au@SiO_2 substrate, illustrating that Au cannot facilitate the hydrogenation of pNTP and hydrogen atoms are unable to migrate through the silica shells.

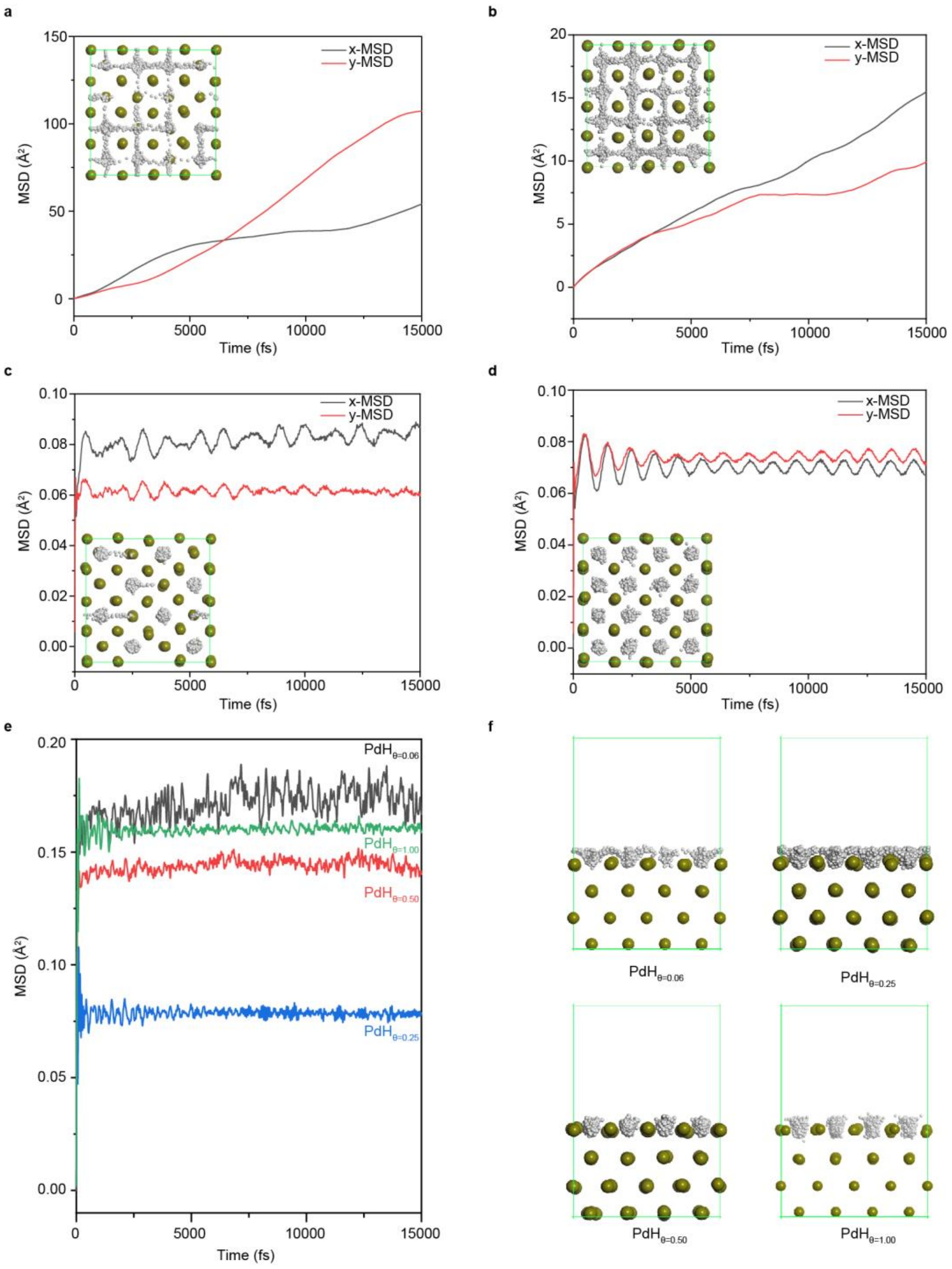

Supplementary Fig. 26 | Mean square distance of $PdH_{\theta=0.06}$, $PdH_{\theta=0.25}$, $PdH_{\theta=0.50}$ and $PdH_{\theta=1.00}$. (**a**) Mean square distance of $PdH_{\theta=0.06}$ along x and y direction. (**b**) Mean square distance of $PdH_{\theta=0.25}$ along x and y direction. (**c**) Mean square distance of $PdH_{\theta=0.50}$ along x and y direction. (**d**) Mean square distance of $PdH_{\theta=1.00}$ along x and y direction. (**e**) Mean square distance of $PdH_{\theta=0.06}$, $PdH_{\theta=0.25}$, $PdH_{\theta=0.50}$ and $PdH_{\theta=1.00}$ along z direction. (**f**) Structures of $PdH_{\theta=0.06}$, $PdH_{\theta=0.25}$, $PdH_{\theta=0.50}$ and $PdH_{\theta=1.00}$ along z direction. Pd: golden; H: grey.

By simulating H atoms with different coverage, we find that H atoms will move quickly and randomly on the surface under the condition of low coverage (Supplementary Fig. 26a). However, with the increase of coverage, the diffusion rate of H atom decreases and most H atoms begin to

vibrate only near their own equilibrium position, indicating that the increase of coverage will lead to the increase of the diffusion energy barrier of H atom on the surface of Pd. In addition, the root mean square displacement in the z direction of H atom with high coverage also increases substantially and tends to spread to the interior of Pd, indicating that the hydrogenation of Pd can only occur under the condition of high coverage. For this reason, the energy barrier of hydrogenation is calculated under the condition of high coverage.

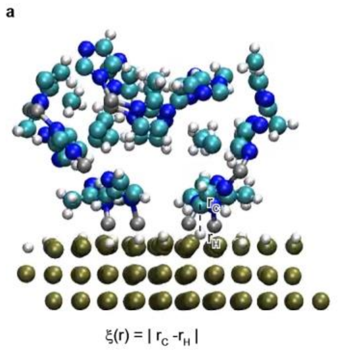

Supplementary Fig. 27 | The illustrations of reaction coordinates. The illustrations of reaction coordinates for the hydrogen atom spillover (**a**) from Pd to ZIF-8 and (**b**) from ZIF-8 back to Pd. Pd: golden; Zn: silver; C: indigo-blue; N: blue; H: grey.

As shown in Supplementary Fig. 27a, we set the distance of C–H as the collective variable (CV) from Pd to ZIF-8. This decrease in C–H distance is implemented to simulate the process of hydrogen spillover. The Z-coordinate of the H atom is established with CV value (Supplementary Fig. 27b), using its diminishing process to simulate the energy barrier for the hydrogen atom moving from ZIF-8 to Pd.

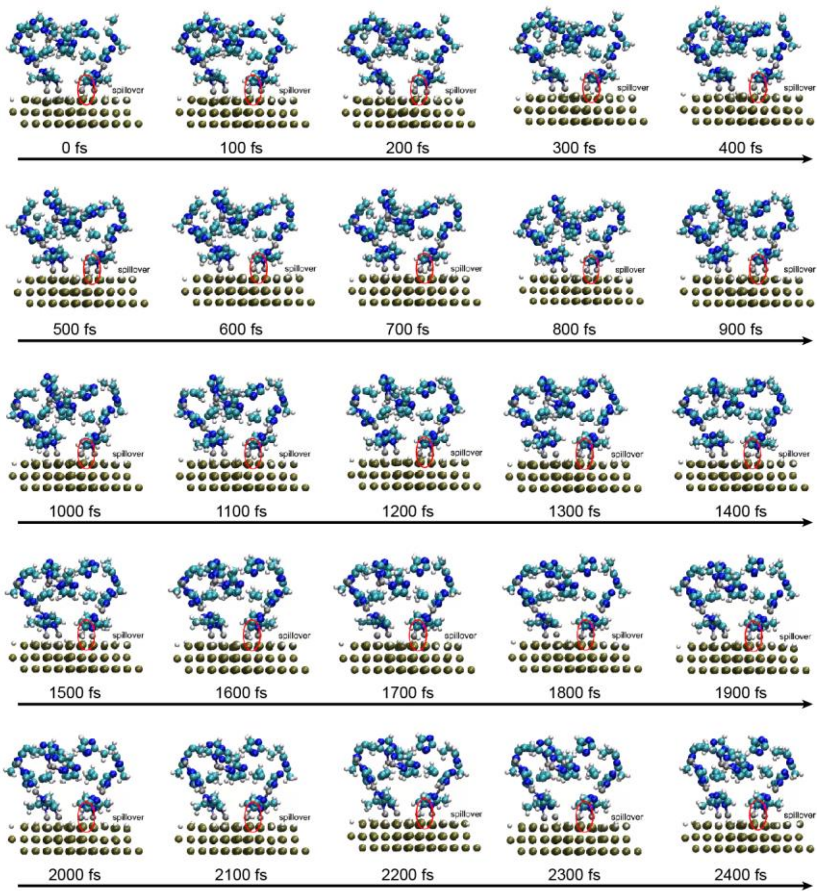

Supplementary Fig. 28 | Snapshots of hydrogen spillover across Pd–ZIF-8 interface from Pd to ZIF-8. Pd: golden; Zn: silver; C: indigo-blue; N: blue; H: grey.

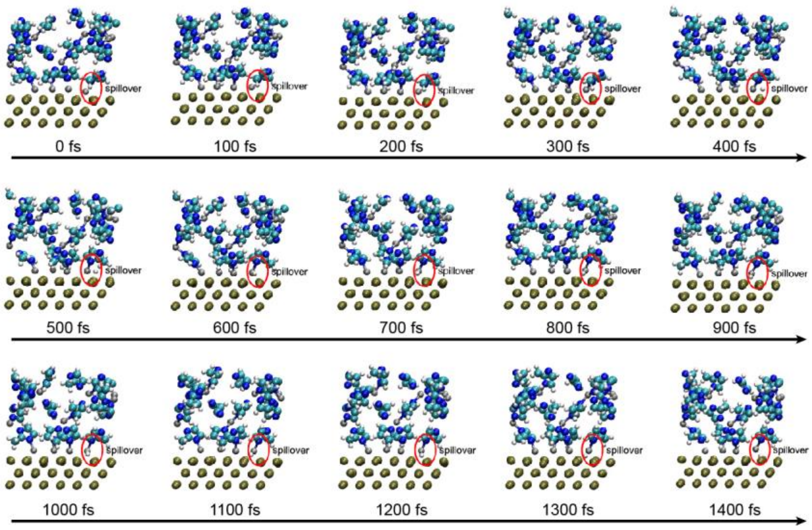

Supplementary Fig. 29 | Snapshots of hydrogen spillover across Pd–ZIF-8 interface from ZIF-8 to Pd. Pd: golden; Zn: silver; C: indigo-blue; N: blue; H: grey.

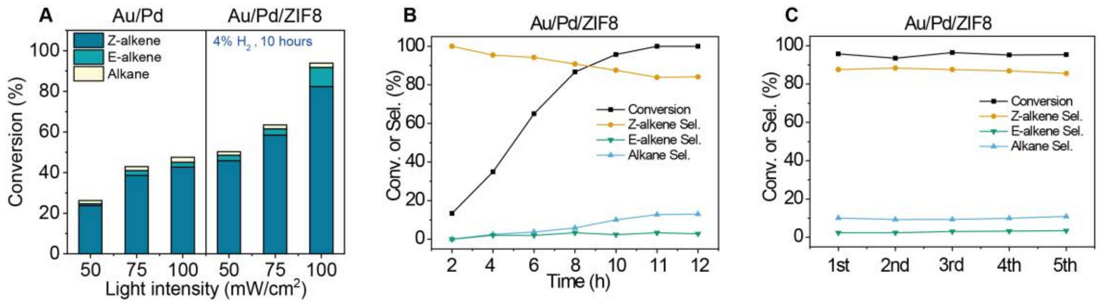

Supplementary Fig. 30 | Catalytic performance of Au/Pd and Au/Pd/ZIF-8 under light irradiation. (**a**) Catalytic performance of Au/Pd and Au/Pd/ZIF-8 in light-driven DAC hydrogenation at various irradiation intensity. (**b**) Time-dependent catalytic performance of Au/Pd/ZIF-8 in light-driven DAC hydrogenation. (**c**) Cycling catalytic performance of Au/Pd/ZIF-8 in light-driven DAC hydrogenation. Each run takes 10 hours. Reaction conditions: catalyst (containing 0.1 mg Au), substrate (DAC, 10 μL), solvent (CH_3CN, 1 mL), 4% H_2 (101 kPa), reaction time (2–12 h).

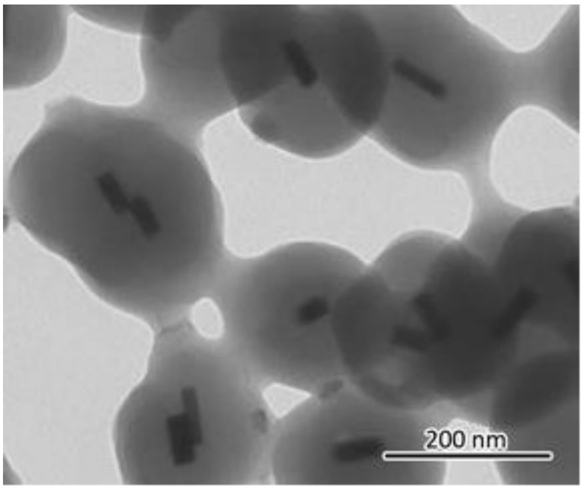

Supplementary Fig. 31 | TEM image of Au NRs@Pd@ZIF8 hybrid structures after catalytic reactions.

TEM image (Supplementary Fig. 31) shows that the morphology and structure of Au NRs@Pd@ZIF8 are well maintained after catalytic reactions.

Supplementary Table 1 | Element content detected by ICP-OES.

Samples	Element content (μg/mL)
Pd NCs	Pd: 13.779
Pd/ZIF-8 for NMR	Pd: 21.166; Zn: 73.468
Pd/ZIF-8 for XRD	Pd: 13.779; Zn: 34.495
Au/Pd	Pd: 3.016; Au: 3.515
Au/Pd/ZIF-8	Pd: 1.048; Au: 1.224; Zn: 2.605

Ten microliters of Pd NC, Au/Pd and Au/Pd/ZIF-8 solutions were dissolved in 1 mL of aqua regia and then diluted to 5 mL with water. A total of 2.52 mg Pd/ZIF-8 for NMR and 1.27 mg Pd/ZIF-8 for XRD were dissolved in 1 mL aqua regia and diluted to 5 mL with water. The metal loading of Pd for Pd/ZIF-8 for NMR is 4.20%, and the metal loading of Pd for Pd/ZIF-8 for XRD is 5.42%.

Supplementary Table 2 | TOF of pNTP hydrogenation of different samples.

**Samples**	**Time (20% conversion)/s**	**TOF /h^{-1}**
Pd/Au	9.12×10^{-3}	2.62×10^{4}
Pd/ZIF-8-20 nm/Au	1.68×10^{-2}	1.42×10^{4}
Pd/ZIF-8-40 nm/Au	3.59×10^{-2}	6.66×10^{3}
Pd/ZIF-8-60 nm/Au	7.14×10^{-2}	3.35×10^{3}
Pd/ZIF-8-120 nm/Au	∞	0

3. Supplementary References

1 Jin, M. et al. Synthesis of Pd nanocrystals enclosed by {100} facets and with sizes <10 nm for application in CO oxidation. *Nano Res.* **4**, 83-91 (2011).

2 Nikoobakht, B. & El-Sayed, M. A. Preparation and Growth Mechanism of Gold Nanorods (NRs) Using Seed-Mediated Growth Method. *Chem. Mater.* **15**, 1957-1962 (2003).

3 Van Hardeveld, R. & Hartog, F. The statistics of surface atoms and surface sites on metal crystals. *Surf Sci.* **15**, 189-230 (1969).

4 Kresse, G. & Furthmüller, J. Efficient iterative schemes for ab initio total-energy calculations using a plane-wave basis set. *Phys. Rev. B* **54**, 11169-11186 (1996).

5 Blöchl, P. E. Projector augmented-wave method. *Phys. Rev. B* **50**, 17953-17979 (1994).

6 Perdew, J. P., Burke, K. & Ernzerhof, M. Generalized Gradient Approximation Made Simple. *Phys. Rev. Lett.* **77**, 3865-3868 (1996).

7 Sprik, M. & Ciccotti, G. Free energy from constrained molecular dynamics. *J. Chem. Phys.* **109**, 7737-7744 (1998).

8 Cheng, T., Xiao, H. & Goddard, W. A. Full atomistic reaction mechanism with kinetics for CO reduction on Cu(100) from ab initio molecular dynamics free-energy calculations at 298 K. *Proc. Natl. Acad. Sci.* **114**, 1795-1800 (2017).

9 Ania, C. O. *et al.* Understanding Gas-Induced Structural Deformation of ZIF-8. *J. Mater. Chem. A.* **3**, 1159-1164 (2012).

10 Liz-Marzán, L. M., Giersig, M. & Mulvaney, P. Synthesis of Nanosized Gold−Silica Core−Shell Particles. *Langmuir* **12**, 4329-4335 (1996).
