# Supplementary material for: Spectroscopic visualization of reversible hydrogen spillover between palladium and metal–organic frameworks toward catalytic semihydrogenation
Source: Nat Commun. 2024 Mar 22;15:2562. doi: 10.1038/s41467-024-46923-3 (PMC10959988; doi:10.1038/s41467-024-46923-3)
Supplement: Supplementary file 3 — Description of Additional Supplementary Files [file 41467_2024_46923_MOESM3_ESM.pdf]

Description of Additional Supplementary Files

File Name: Supplementary Movie 1
Description: **The movie of hydrogen spillover across Pd–ZIF-8 interface from Pd to ZIF-8.** Pd: golden; Zn: silver; C: indigo-blue; N: blue; H: grey.

File Name: Supplementary Movie 2
Description: **The movie of hydrogen spillover across Pd–ZIF-8 interface from ZIF-8 to Pd.** Pd: golden; Zn: silver; C: indigo-blue; N: blue; H: grey.
